# High breakdown voltage in AlGaN/GaN HEMTs using AlGaN/GaN/AlGaN quantum-well electron-blocking layers

**DOI:** 10.1186/1556-276X-9-433

**Published:** 2014-08-27

**Authors:** Ya-Ju Lee, Yung-Chi Yao, Chun-Ying Huang, Tai-Yuan Lin, Li-Lien Cheng, Ching-Yun Liu, Mei-Tan Wang, Jung-Min Hwang

**Affiliations:** 1 Institute of Electro-Optical Science and Technology, National Taiwan Normal University, 88, Sec. 4, Ting-Chou Road, Taipei 116, Taiwan; 2 Institute of Electronics Engineering, National Taiwan University, 1, Sec. 4, Roosevelt Road, Taipei 106, Taiwan; 3 Institute of Optoelectronic Sciences, National Taiwan Ocean University, 2, Pei-Ning Road, Keelung 202, Taiwan; 4 Solid-State Lighting Systems Department, Green Energy and Environment Research Laboratories, Industrial Technology Research Institute (ITRI), Hsinchu 310, Taiwan

**Keywords:** AlGaN/GaN HEMT, 2-DEG, Breakdown voltage

## Abstract

In this paper, we numerically study an enhancement of breakdown voltage in AlGaN/GaN high-electron-mobility transistors (HEMTs) by using the AlGaN/GaN/AlGaN quantum-well (QW) electron-blocking layer (EBL) structure. This concept is based on the superior confinement of two-dimensional electron gases (2-DEGs) provided by the QW EBL, resulting in a significant improvement of breakdown voltage and a remarkable suppression of spilling electrons. The electron mobility of 2-DEG is hence enhanced as well. The dependence of thickness and composition of QW EBL on the device breakdown is also evaluated and discussed.

## Background

GaN-based high-electron-mobility transistors (HEMTs) have attracted considerable interests for the high-speed and high-power-switching applications because of their outstanding electronic properties. The high sheet-carrier density of the two-dimensional electron-gas (2-DEG) [1,2] and large critical breakdown electric field [3,4] allow the fabricated HEMT devices with unprecedented high drain current density and large breakdown voltage, which are essential for the important applications of power devices [5-9]. However, the high sheet electron density inherently in GaN-based HEMTs will inevitably induce the spillover of transport electrons at high-drain-voltage conditions, and that becomes a growing issue. In general, the confinement of transport electrons to the bottom side of the device is insufficient in the conventional AlGaN/GaN HEMT, due mainly to the insufficient potential height provided by the GaN buffer layer underneath. Consequently, transport electrons supposed to be confined within the 2-DEG channel would easily spill or leak into the buffer layer, causing a rapid increase of subthreshold drain leakage currents, accelerating the device breakdown. The above-mentioned phenomenon is often interpreted as the ‘punchthrough effect,’ hindering the further applications of GaN-based HEMTs. Therefore, methods improving the confinement of transport electrons within the channel layer and alleviating the punchthrough effect are necessary. Over the years, several approaches, such as the introduction of p-type doping to the GaN buffer layer [10-12] and the use of AlGaN/GaN/AlGaN double-heterojunction HEMTs [13-15], have been reported to enhance the breakdown voltage of GaN-based HEMTs. The basic principle is to raise the conduction band of the GaN buffer layer, and thus generates a deeper and narrower potential well for the better confinement of 2-DEG.

In this work, we present an improved bottom confinement of 2-DEG by introducing the AlGaN/GaN/AlGaN quantum-well (QW) electron-blocking layer (EBL) structure. It is shown that the large electric field induced at the interfaces of AlGaN/GaN/AlGaN QW EBL effectively depletes the spilling electrons toward the 2-DEG channel. As compared to previous approaches, the subthreshold drain leakage current becomes less sensitive to the drain voltage (*V*_ds_), and that postpones the HEMT breakdown. Meanwhile, our proposed structure not only exhibits the highest electron mobility among other compared HEMT devices but also allows a great tolerance for epitaxial imperfections during the device fabrication. As a result, we conclude that the proposed AlGaN/GaN/AlGaN QW EBL HEMT is viable and highly promising for the high-speed and high-power-switching applications.

## Methods

**Figure 1:**
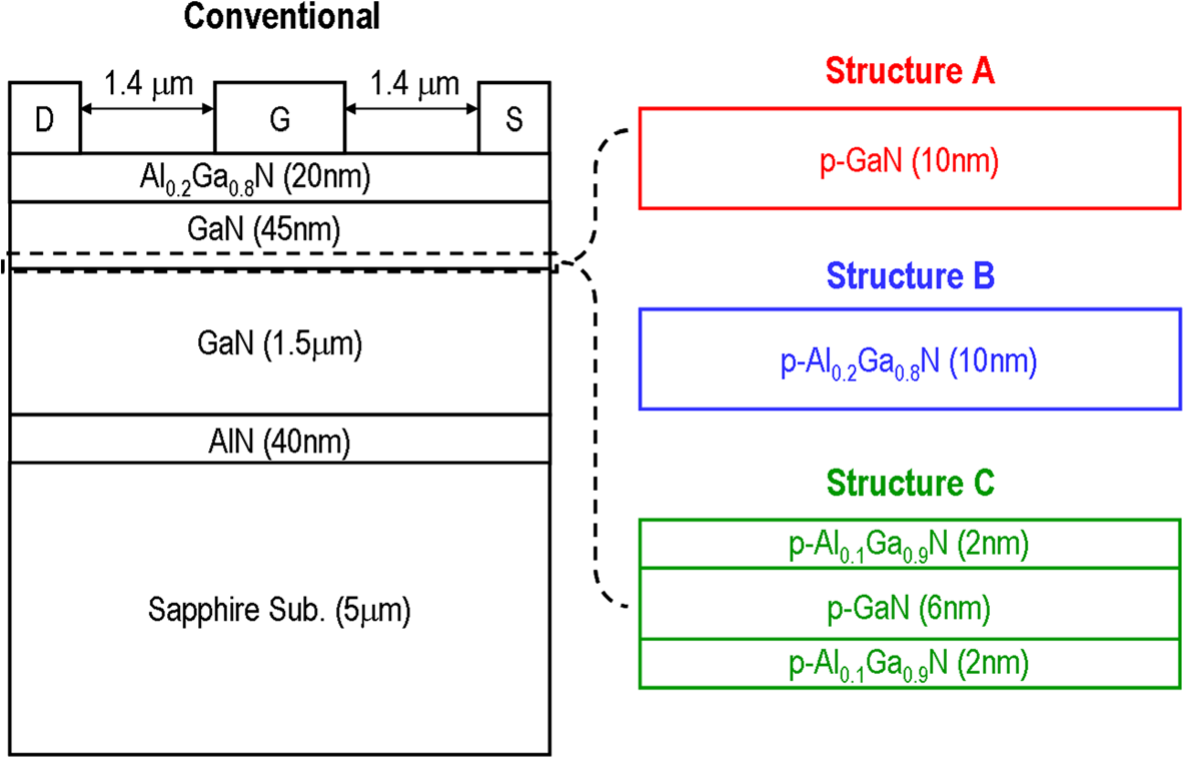
**A schematic of the conventional AlGaN/GaN HEMT.** For structure A, a 10-nm-thick EBL with p-type polarity (*p* = 1 × 10^18^ cm^−2^) was inserted. For structure B and structure C, the original 10-nm-thick GaN EBL was replaced with Al_0.1_Ga_0.9_N EBL and Al_0.1_Ga_0.9_N/GaN/Al_0.1_Ga_0.9_N QW EBL, respectively.

For comparison, four types of devices were numerically studied and the schematic structures are plotted in Figure 1. All devices are designed on an insulating sapphire substrate and have a 40-nm-thick AlN nucleation layer followed by an un-doped GaN buffer layer with a thickness of 1.5 μm.

For the conventional HEMT, a 45-nm-thick un-doped GaN was employed as the channel layer. To alleviate the 2-DEG spillover, a 10-nm-thick EBL was created by p-type doping (*p* = 1 × 10^18^ cm^−3^) to the bottom region of the GaN channel layer, i.e., structure A. For structure B and structure C, we replaced the original 10-nm-thick GaN EBL with Al_0.1_Ga_0.9_N EBL and Al_0.1_Ga_0.9_N/GaN/Al_0.1_Ga_0.9_N QW EBL, respectively. The dopant polarity and doping concentration for the EBLs of structure B and structure C remain the same as *p* = 1 × 10^18^ cm^−3^. Finally, all structures were capped by an un-doped 20-nm-thick Al_0.2_Ga_0.8_N barrier layer. The HEMT dimension is designed as 5.4 μm × 200 μm with a gate length of 0.6 μm for numerical analyzing. Both gate-source and gate-drain distances were set to 1.4 μm. To reduce the complexity of physical simulation of the device, here, we assume that the source and drain metals are the perfect Ohmic contact to the Al_0.2_Ga_0.8_N barrier layer, and the gate metal is the ideal Schottky contact. To calculate the performance of the HEMT, we have used the finite element simulation program - APSYS. The electrical property of the HEMT was performed by solving the Poisson's equation and the continuity equation. The transport model of electrons and holes considers their drift and diffusion in the devices. The material parameters used in this work can be found in [16] and the references therein. The bandgap of Al_*x*_Ga_1 − *x*_N as a function of the aluminum composition (*x*) is given by

(1) $$E_{g}\left(x\right)=6.13x+3.42\left(1-x\right)-1.20x\left(1-x\right)\;\mathrm{eV}\text{.}$$

The bowing factor adopted in Equation 1 is *b* = 1.20 eV [17], and the conduction band offset for AlGaN/GaN heterojunction is set to 0.68. The APSYS program employs the 6 × 6 *k* · *p* model to depict the energy band profile for the strained wurtzite structure [18-20]. Both spontaneous and piezoelectric polarizations were considered in the simulations. The spontaneous polarization in c-plane Al_*x*_Ga_1 − *x*_N as a function of aluminum composition (*x*) is given by [21]

(2) $$P_{{\mathrm{Al}}_{x}{\mathrm{Ga}}_{1\;-\;x}N}^{\mathrm{sp}}\left(x\right)=\left[-0.090x-0.034\left(1-x\right)+0.019x\left(1-x\right)\right]\;{\mathrm{cm}}^{-2}\text{,}$$

while the piezoelectric polarization of AlGaN pseudomorphically grown on the GaN template is calculated by [22]

(3) $$P_{{\mathrm{Al}}_{x}{\mathrm{Ga}}_{1\;-\;x}N}^{\mathrm{pz}}\left(x\right)=\left[-0.0525x-0.0282x\left(1-x\right)\right]{\mathrm{cm}}^{-2}\text{.}$$

In the drift-diffusion simulations of AlGaN/GaN HEMTs, the value of electron mobility is critical to describe the transport behavior of 2-DEG. The electron mobility as a function of the longitudinal electric field in the 2-DEG channel, *μ*_*n*_(*E*), is assumed to follow the Caughey and Thomas model given by [23]

(4) $$\mu_{n}\left(E\right)=\mu_{n0}{\left[1+\frac{\mu_{n0}E}{v_{\mathrm{sat}}}\right]}^{-1/\beta_{n}}\;{\mathrm{cm}}^{2}/V‒s$$

where *μ*_*n*0_ is the low-field electron mobility, *ν*_sat_ is the saturated value of the electron velocity, and *β*_*n*_ is a fitting parameter. To increase the accuracy of the calculation for the breakdown voltage and near-breakdown behavior of the HEMT, it is necessary to include the impact ionization. The generation rate of electron-hole pairs due to impact ionization is given by [24]

(5) $$G=\alpha_{n}\frac{J_{n}}{q}+\alpha_{p}\frac{J_{p}}{q}$$

where *α*_*n*_ and *α*_*p*_ are the electron and hole ionization rate defined as the number of electron-hole pairs generated by an electron per unit distance traveled, and *J*_*n*_ and *J*_*p*_ are the current density for electrons and holes, respectively. Both *α*_*n*_ and *α*_*p*_ are strongly dependent on the electric field applied on the device and can be expressed as [25]

(6) $$\alpha_{n,p}={\alpha^{\infty }}_{n,p}\exp\left[-\left(\frac{{E^{\mathrm{crit}}}_{n,p}}{E}\right)\right]\text{.}$$

Specifically, to calculate the impact ionization in the GaN wurtzite structure, the values of coefficients *α*_*n*,*p*_ and *E*^cri*t*^_*n*,*p*_ were set to be 2.60 × 10^8^ cm^−1^ and 3.42 × 10^7^ V cm^−1^ for electrons, and 4.98 × 10^6^ cm^−1^ and 1.95 × 10^7^ V cm^−1^ for holes, respectively.

## Results and discussion

**Figure 2:**
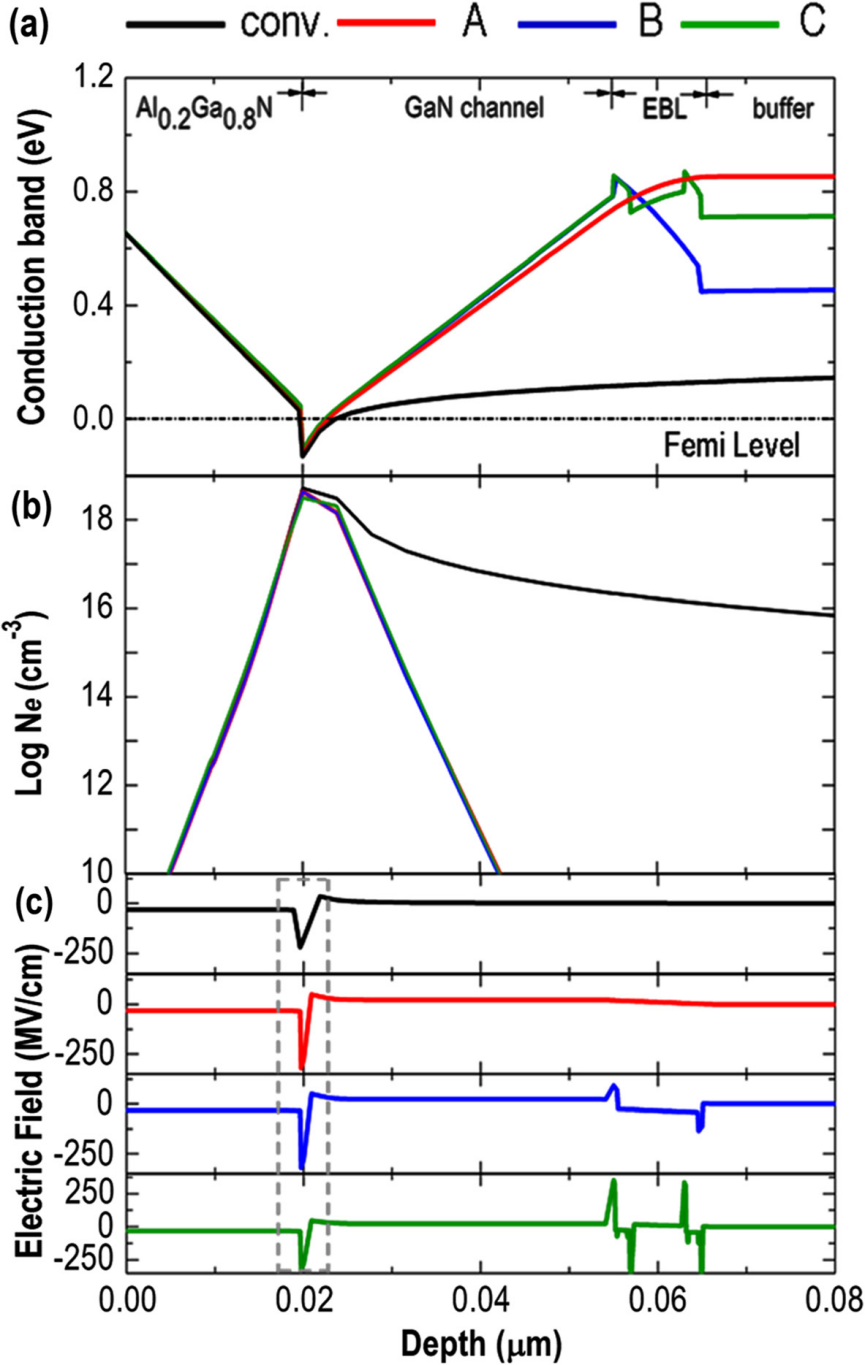
**Conduction band, electron density, and electric field distribution versus depth plots. (a)** Calculated conduction band profiles of all devices under the neutral bias condition. **(b)** Distribution of three-dimensional electron density (*N*_e_) in a semi-log scale for all devices. **(c)** Corresponding electric field distributed over all devices. The dotted-line rectangle marks the region where the 2-DEG channel belongs.

Figure 2a shows a comparison of calculated conduction band profiles for all devices in the neutral bias condition. As observed on the conventional AlGaN/GaN HEMT (black solid line), the potential height toward the GaN buffer layer is insufficient to well confine the 2-DEG, and a spillover of transport electrons is hence expected under high-drain-voltage conditions. However, such phenomenon is alleviated in structures A to C, as a deeper and narrower potential well is formed to serve as the 2-DEG channel, providing a better confinement of transport electrons. Figure 2b plots the distribution of three-dimensional electron density (*N*_e_) in a semi-log scale for all devices. Accordingly, *N*_e_ of structures A to C exhibits an almost identical distributed profile and have a similar peak value of *N*_e_ = 4.24 × 10^18^ cm^−3^. Most importantly, introducing the EBL effectively reduces the spillover of transport electrons as the *N*_e_ (at depth = 0.04 μm) is remarkably decreased from *N*_e_ = 7.21 × 10^16^ cm^−3^ (the conventional HEMT) to *N*_e_ = 1.48 × 10^11^ cm^−3^ (structures A to C). Such orders-of-magnitude reduction in *N*_e_ indicates a significant enhancement of 2-DEG confinement beneficial from the employment of EBL structures. The origin of the above observations can be further illustrated by inspecting the corresponding distributed electric field (Figure 2c). For the conventional AlGaN/GaN HEMT, a negative electric field is induced in the 2-DEG channel (marked by the dotted-line rectangle) due to the accumulation of polarization charges supported by the Al_0.2_Ga_0.8_N barrier layer. The electric field becomes positive in the region below the 2-DEG channel. Therefore, it is beneficial to repel the transport electrons toward the 2-DEG channel, confining them and preventing punchthrough. However, the magnitude of the electric field is generally too small to repel the spilling electrons in the conventional AlGaN/GaN HEMT structure. In contrast, the magnitude of the electric field is considerably enhanced by intentionally inserting the EBL into the HEMT, especially for structure C. Obviously, an extremely large electric field of *E* = 350 MV/cm is induced in structure C (at the bottom side of GaN channel layer, depth approximately 0.055 μm), which effectively depletes and confines the transport electrons into the 2-DEG channel, and that subsequently suppresses the subthreshold drain leakage current.

**Figure 3:**
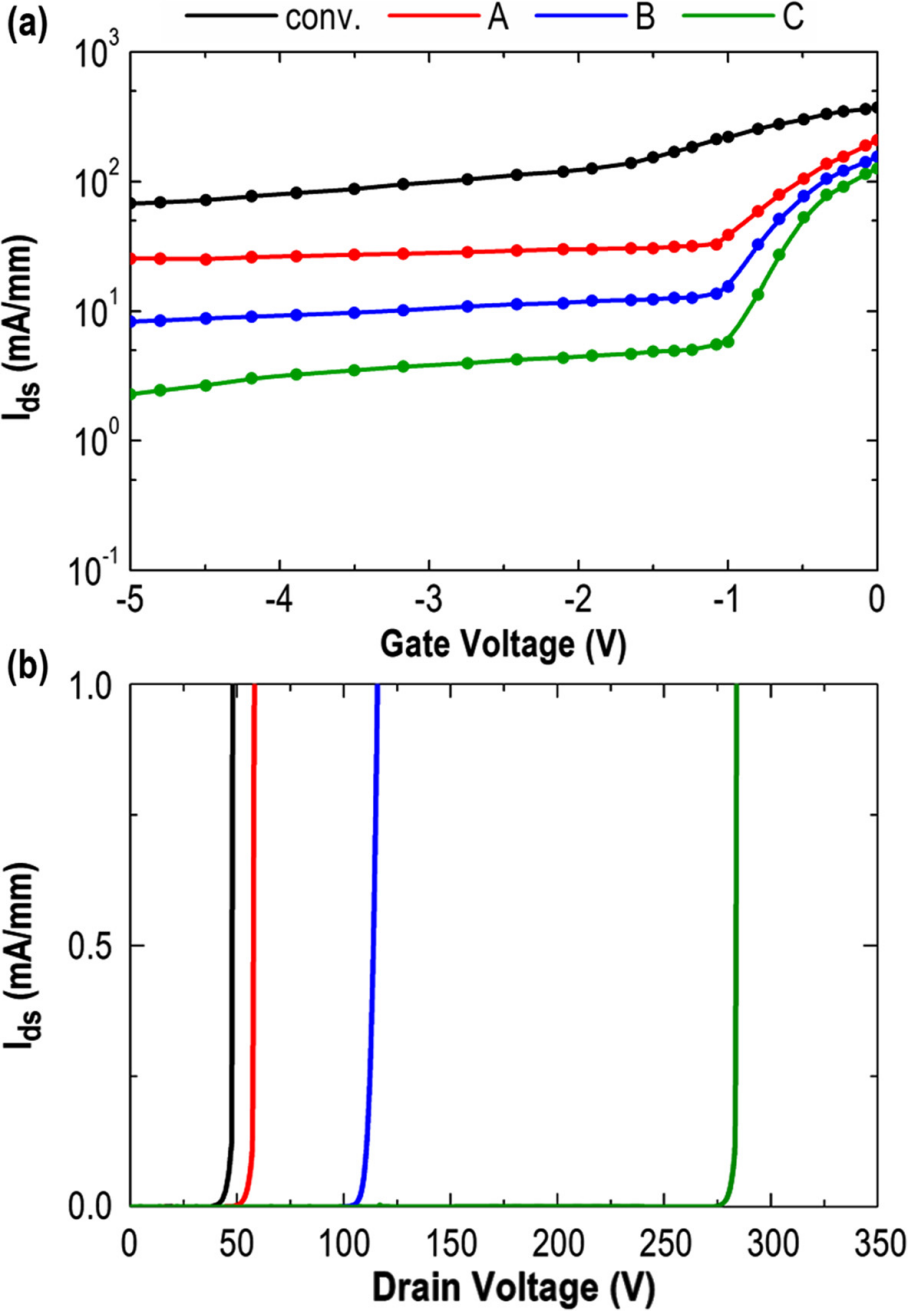
**DC transfer characteristics and subthreshold drain leakage versus drain voltage plots. (a)** Transfer characteristics (*I*_ds_ vs. *V*_g_) for all devices with a drain voltage of *V*_ds_ = 30 V. **(b)** Subthreshold drain leakage current as a function of drain bias for all devices under a closed-gate condition of *V*_g_ = −5 V.

Figure 3a shows DC transfer characteristics, i.e., drain current (*I*_ds_) versus gate voltage (*V*_g_), of all devices in a semi-log scale with a drain voltage (*V*_ds_) of *V*_ds_ = 30 V. At a given value of *V*_g_, the conventional AlGaN/GaN HEMT always shows the largest subthreshold drain leakage current, and that is obviously decreased in structures A to C. While supplying a sufficiently high *V*_ds_ on the conventional AlGaN/GaN HEMT, the transport electrons can directly bypass the gate depletion region and drift into the GaN buffer layer underneath, increasing the subthreshold drain leakage current even under the threshold gate voltage (*V*_th_) operation. Clearly, structure C exhibits the lowest subthreshold drain leakage current among all devices. It indicates that the transport electrons are effectively blocked by the AlGaN/GaN/AlGaN QW EBL and thus are not able to migrate via the buffer layer and contribute the leakage current. Figure 3b shows the subthreshold drain leakage versus drain voltage at a closed-gate condition below a threshold bias of *V*_g_ = −5 V for all devices. Here, the breakdown voltage (*V*_br_) of the HEMT is defined as the voltage at which the subthreshold drain leakage current increases superlinearly with the drain voltage. The breakdown voltage identified for the conventional AlGaN/GaN HEMT, structure A, structure B, and structure C are *V*_br_ = 48 V, *V*_br_ = 58 V, *V*_br_ = 115 V, and *V*_br_ = 285 V, respectively. Restated, among all devices, a dramatic enhancement of *V*_br_ and a large reduction of subthreshold drain leakage current in structure C are mainly attributed to its improved confinement of transport electrons by the AlGaN/GaN/AlGaN QW EBL.

**Figure 4:**
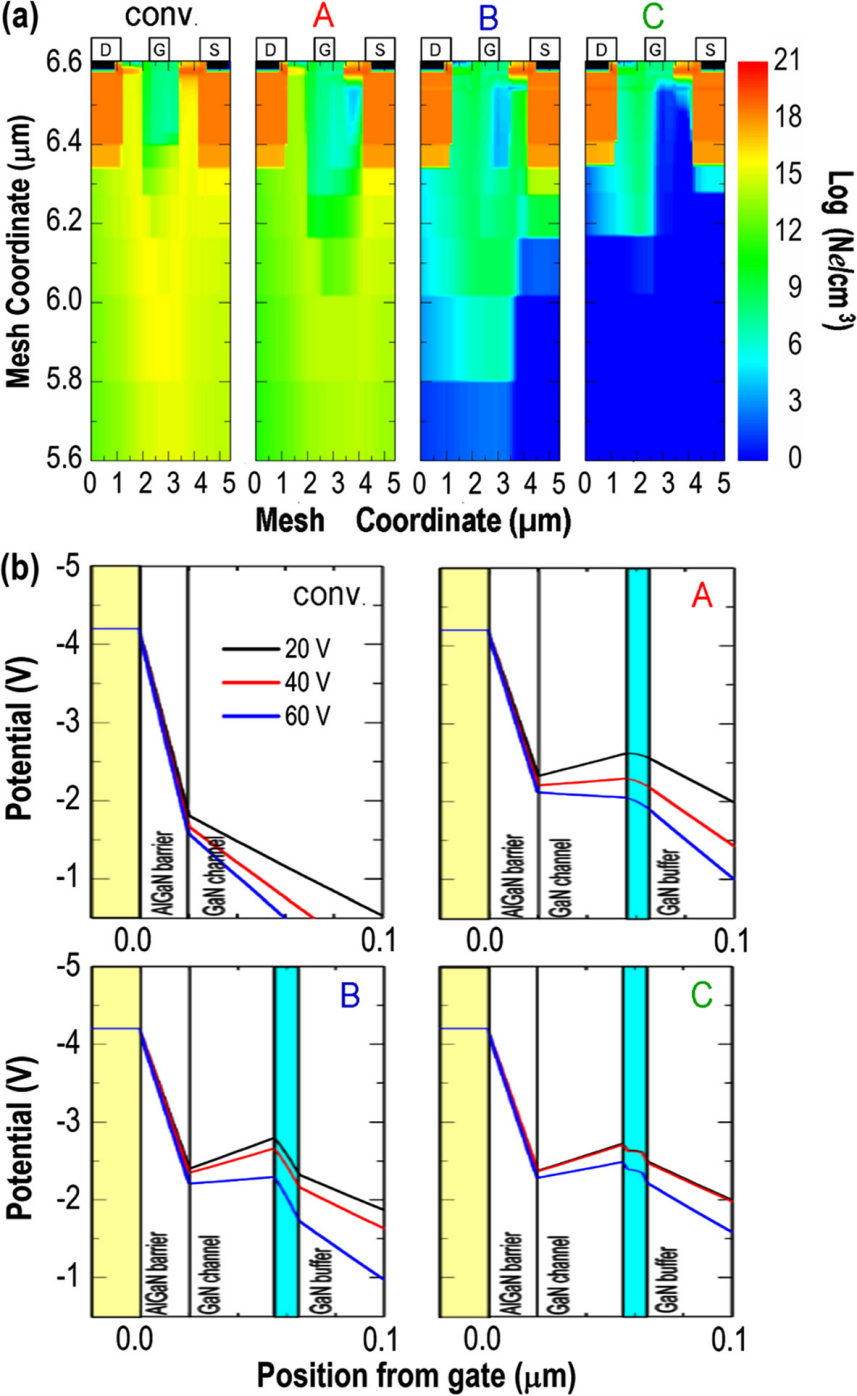
**Cross sections of the electron concentration distribution at a closed-gate condition and cross-sectional potential profiles. (a) ***N*_e_ distributions in all devices at a closed-gate condition of *V*_g_ = −5 V and *V*_ds_ = 80 V. **(b)** Cross-sectional potential profiles for all devices, where *V*_g_ = −5 V, *V*_ds_ = 20 V (black line), *V*_ds_ = 40 V (red line), and *V*_ds_ = 60 V (blue line). The EBL region is marked by the light-blue rectangle.

Figure 4a plots cross sections of the electron concentration distribution at a closed-gate condition of *V*_g_ = −5 V and *V*_ds_ = 80 V for all devices. Obviously, the electrons under the gate electrode are depleted completely by the gate-induced electric field in the conventional AlGaN/GaN HEMT. Nevertheless, as the potential height provided by the GaN buffer layer is small, most of the transport electrons can still bypass this depletion region by migrating across the GaN buffer layer to the lower potential regions, causing an inevitable subthreshold drain leakage current. In structures A to C, the potential height (toward the GaN buffer layer) created by the EBL is increased, which prevents the transport electrons from spilling into the GaN buffer layer, reducing the HEMT's subthreshold drain leakage current. The functionality of EBL is further examined by inspecting the cross-sectional potential profiles for all devices under a closed-gate condition of *V*_g_ = −5 V with *V*_ds_ increasing from *V*_ds_ = 20 V to *V*_ds_ = 60 V in 20-V interval (Figure 4b). Accordingly, for the conventional AlGaN/GaN HEMT, there is already no potential barrier toward the GaN buffer layer even operating at the low drain bias of *V*_ds_ = 20 V. The situations become worse for the higher-drain-bias conditions of *V*_ds_ = 40 V and *V*_ds_ = 60 V. Thus, it is the main reason responsible for the smallest *V*_br_ of the conventional AlGaN/GaN HEMT. In contrast, introducing the EBL can raise the conduction band of the GaN channel layer by the bandgap difference, building a deeper potential well to confine 2-DEG, preventing punchthrough. Such effect is noticeable in structure C even when the HEMT is operated under a high-drain-bias condition. Additionally, due to the large electric field induced at the interfaces of AlGaN/GaN/AlGaN QW EBL, the potential decline of structure C in the conduction band (marked by the light-blue rectangle) with the increasing of *V*_ds_ is less pronounced, considerably postponing the device breakdown.

**Figure 5:**
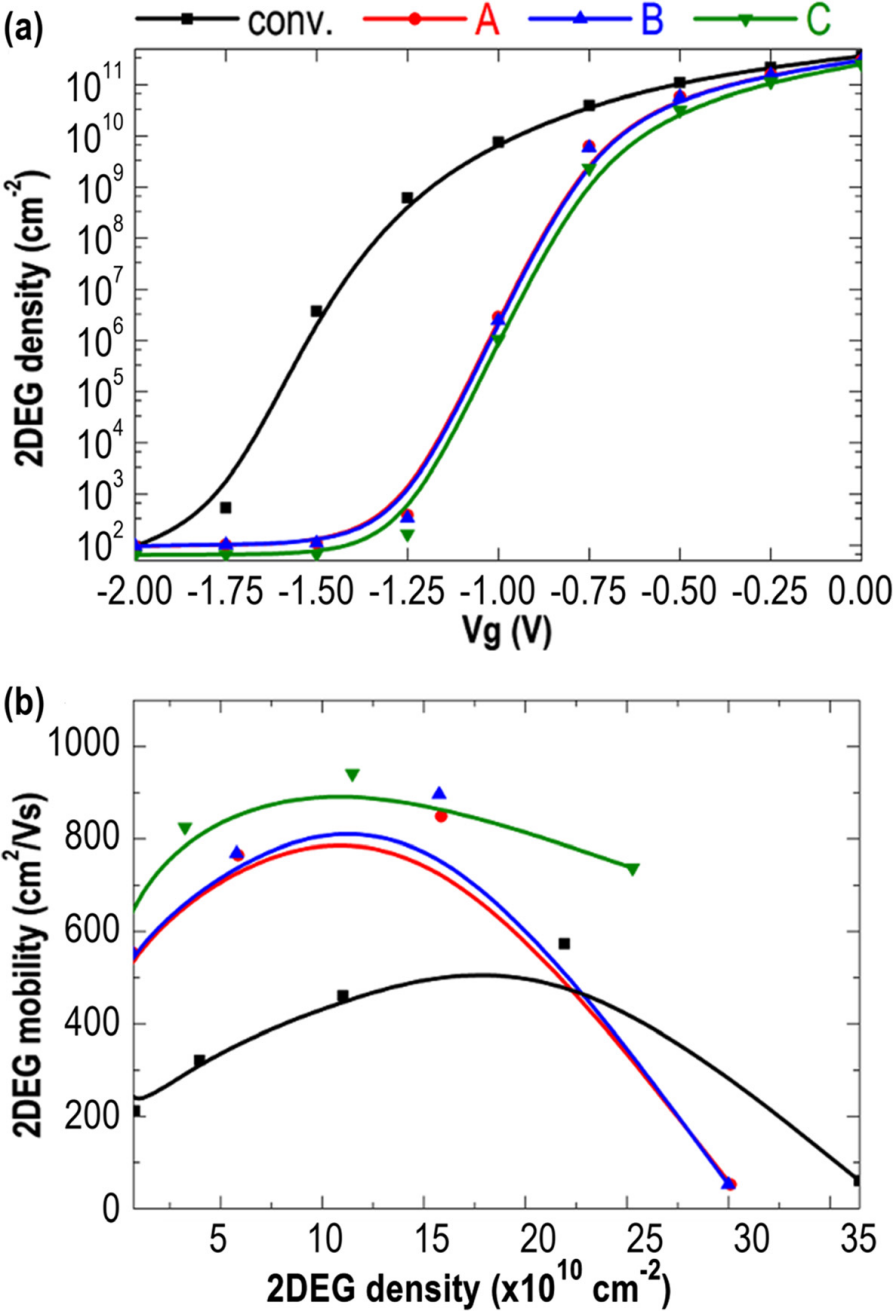
**Dependence of 2-DEG density on gate voltage and 2-DEG mobility (*μ*) versus 2-DEG density plots. (a)** Dependence of 2-DEG density on gate voltage (*V*_g_) and **(b)** 2-DEG mobility (*μ*) versus 2-DEG density for all devices.

Figure 5a plots the 2-DEG density as a function of *V*_g_ for all devices. As compared to structures A to C, the conventional AlGaN/GaN HEMT has to be supplied with a much larger negative gate voltage to close the 2-DEG channel and diminish the 2-DEG density to a background value of approximately 10^2^ cm^−2^. Additionally, the estimated slope of the conventional AlGaN/GaN HEMT (i.e., the difference of 2-DEG density divided by the difference of *V*_g_) is not as steep as that of structures A to C, suggesting a weak confinement of transport electrons. However, the 2-DEG density of structures A to C increases rapidly at a low gate voltage (−1.25 V ≤ *V*_g_ ≤ −0.50 V), and that becomes saturated to approximately 10^11^ cm^−2^ at higher *V*_g_. Figure 5b shows the 2-DEG mobility (*μ*) versus 2-DEG density for all devices. The 2-DEG mobility of all devices initially increases along with the increasing of 2-DEG density, primarily attributed to the enhancement of the screening effect against the ionized ion scattering [25-27]. Yet, the mobility degrading with the further increase of 2-DEG density is considered to be the result of electrons spilling into the AlGaN barrier layer [28-30]. Most importantly, structure C always exhibits the highest electron mobility and achieves a maximum value of *μ* = 940 cm^2^/V-s. Such high electron mobility is critical for the high-speed and high-power-switching applications.

**Figure 6:**
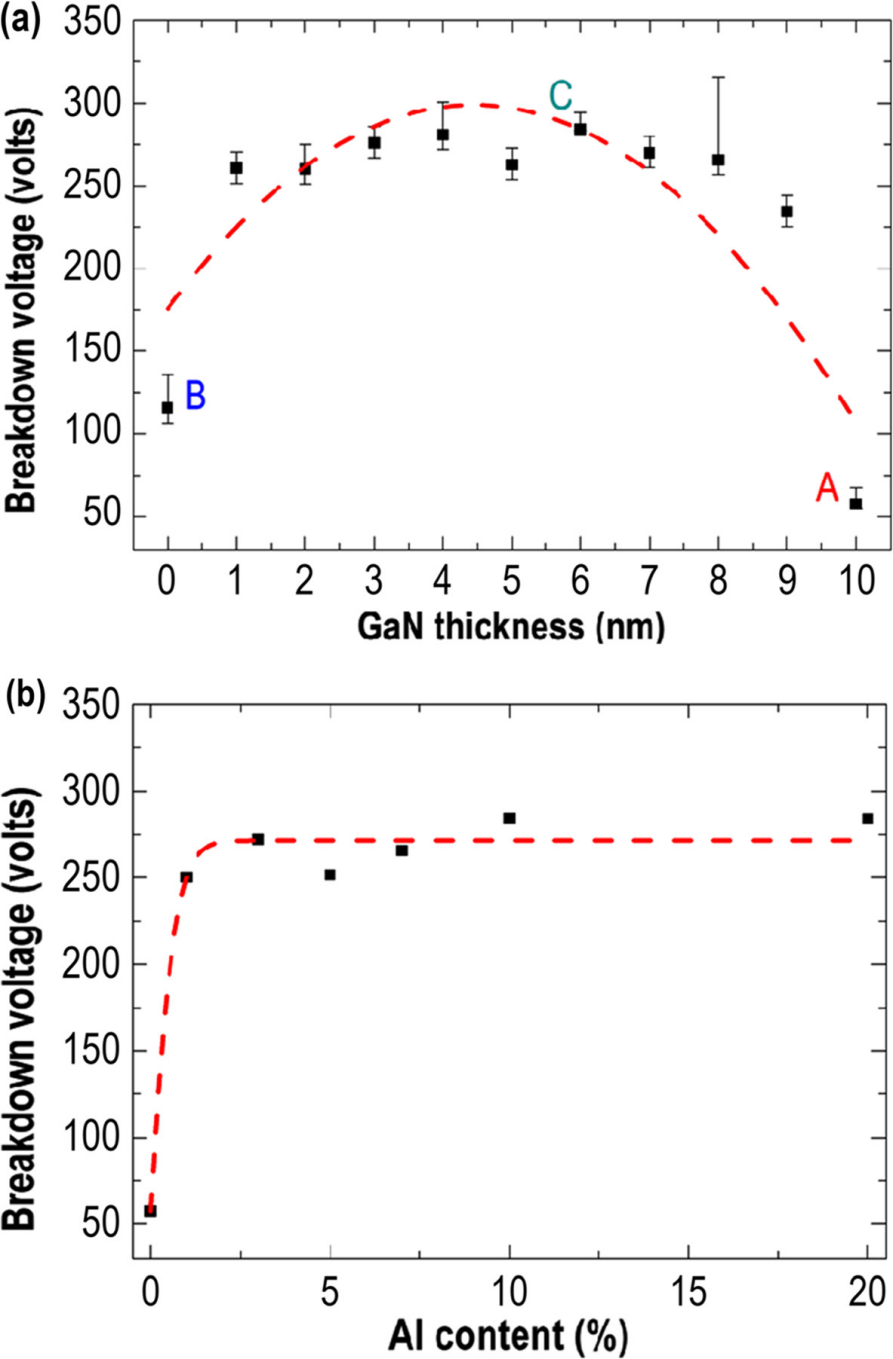
**Breakdown voltage versus GaN thickness and dependence of aluminum composition on breakdown voltage. (a)** HEMT's breakdown voltage versus the GaN thickness of QW EBL, where the barrier layer of QW EBL is Al_0.1_Ga_0.9_N and the total thickness of QW EBL is set to 10 nm. **(b)** Dependence of aluminum composition of QW EBL on the HEMT's breakdown voltage, where the GaN thickness of QW EBL is set to 6 nm and the total thickness of QW EBL is again fixed to 10 nm.

Finally, we are going to discuss the dependence of thickness and composition of QW EBL on the breakdown voltage of the HEMT. Figure 6a plots the breakdown voltage versus the GaN thickness of QW EBL, where the barrier layer of QW EBL is Al_0.1_Ga_0.9_N, and the total thickness of QW EBL is set to 10 nm. As compared to structure A (entire 10-nm-thick GaN EBL) and structure B (entire 10-nm-thick Al_0.1_Ga_0.9_N EBL), introducing the QW EBL considerably enhances the breakdown voltage to a much higher level with an average value of *V*_br_ = 250 V. The ideal GaN thickness of QW EBL is around 4 to 6 nm, which provides a sufficient space to accommodate spilling electrons, prohibiting the further leakage of transport electrons into the GaN buffer layer. Figure 6b shows the dependence of aluminum composition of QW EBL on the breakdown voltage, where the GaN thickness is set to 6 nm, and the total thickness of QW EBL is again fixed to 10 nm. Clearly, the breakdown voltage only fluctuates slightly away from the line of *V*_br_ = 250 V while increasing the aluminum composition of the QW EBL from Al = 3% to Al = 20%, offering a greater tolerance for epitaxial imperfections during the fabrication of a AlGaN/GaN/AlGaN QW EBL structure.

## Conclusions

In conclusion, we propose a novel AlGaN/GaN/AlGaN QW EBL structure to alleviate the punchthrough effect that is generally observed on the conventional AlGaN/GaN HEMT. The introduction of AlGaN/GaN/AlGaN QW EBL leads to a better confinement of transport electrons into the 2-DEG channel, resulting in a reduction of subthreshold drain leakage current and a postponement of device breakdown. The large electric field induced at the interfaces of AlGaN/GaN/AlGaN QW EBL, which effectively depletes the spilling electrons toward the 2-DEG channel, is mainly responsible for the improved performances.

## Competing interests

The authors declare that they have no competing interests.

## Authors’ contributions

Y-CY, L-LC, and C-YL carried out the simulation program and participated in the design of the study. C-YH and T-YL carried out the calculation and helped to draft the manuscript. M-TW and J-MH participated in the design of the study. Y-JL conceived the study and participated in its design and coordination and helped to draft the manuscript. All authors read and approved the final manuscript.
